# Directionality of longitudinal associations between frontostriatal structural connectivity and depressive symptoms in adolescent girls

**DOI:** 10.1111/jcpp.70127

**Published:** 2026-03-05

**Authors:** Marjolein E.A. Barendse, Chris J. Machle, Rogier Kievit, Jennifer H. Pfeifer

**Affiliations:** ^1^ Department of Child and Adolescent Psychiatry/Psychology Erasmus Medical Center Rotterdam The Netherlands; ^2^ Department of Psychology University of Oregon Eugene Oregon USA; ^3^ Donders Center for Medical Neurosciences Radboud University Medical Center Nijmegen The Netherlands

**Keywords:** Reward, white matter, depression, puberty, age

## Abstract

**Background:**

Individual differences in the structure and function of the frontostriatal reward network have been related to depression. However, there is a strong need for prospective, longitudinal studies aiming to understand the role of frontostriatal networks in depression in a developmental context. We aimed to examine bidirectional associations between structural connectivity in the frontostriatal reward network and depressive symptoms in adolescent girls, as well as to determine to what extent the directionality and strength of these associations are dependent on age or pubertal stage.

**Methods:**

About 596 observations from 174 adolescent girls (up to 4 time points per person, ages 10–17) were included. Depressive symptoms were measured with the Center for Epidemiological Studies‐Depression scale for Children and pubertal stage with the Pubertal Development Scale and the Tanner Stage Line Drawings. Probabilistic tractography was done on diffusion‐weighted imaging scans to obtain average fractional anisotropy from ventral striatum to orbitofrontal cortex and ventral striatum to ventromedial prefrontal cortex tracts.

**Results:**

Linear mixed‐effects models showed that frontostriatal connectivity was not associated with subsequent change in depressive symptoms. Depressive symptoms were also not associated with subsequent change in frontostriatal connectivity. Depressive symptoms increased with age and pubertal stage, but the association with connectivity did not vary with age or pubertal stage.

**Conclusions:**

This suggests previously reported cross‐sectional associations might not pertain to developmental effects in girls. Future research should examine prospective associations between frontostriatal functional connectivity and depression.

## Introduction

It is well known that the prevalence of depression increases strongly as children transition into adolescence (Costello, Copeland, & Angold, 2011). Rates of depressive symptoms in adolescents have also gone up in recent years in western countries and are much higher in girls compared with boys (Keyes, Gary, O'Malley, Hamilton, & Schulenberg, 2019). Adolescent mental health has important impacts on several adult outcomes, including adult mental health, educational attainment, and employment prospects (Clayborne, Varin, & Colman, 2019), demonstrating the importance of identifying risk factors during this developmental period.

Brain developmental variables have been considered as potential risk factors. Adolescence is a time of substantial neural changes, including increases in white matter volume, which are for an important part driven by myelination and increases in axonal diameter (Corrigan et al., 2021; Lebel & Deoni, 2018; Paus, 2010). These changes are partially ‘expected’, resulting from genetic processes and pubertal hormonal changes, but also partially experience‐dependent (Pfeifer & Allen, 2021).

A key brain network of interest in the context of depression is the frontostriatal reward network, which entails connections between the ventral striatum and medial prefrontal regions. The typical development of this network during adolescence includes increasing ventral striatal activation to rewards (Braams, van Duijvenvoorde, Peper, & Crone, 2015), thinning of the prefrontal cortex, and decreasing volume of the nucleus accumbens (Wierenga et al., 2018). White matter studies show increasing volume and strengthening microstructure in adolescence (Lebel & Deoni, 2018; Peters et al., 2012), but they did not study the frontostriatal connections specifically. At the neurochemical level, sex hormones drive increases in dopamine synthesis and changes in its action that depend on brain region (Sinclair, Purves‐Tyson, Allen, & Weickert, 2014).

There is evidence for an association between blunted striatal reward response and later development of depression in adolescents (Toenders et al., 2019), in line with findings of blunted striatal activation and hyperactivation of the orbitofrontal cortex (OFC) in response to rewards in adults with depression (Ng, Alloy, & Smith, 2019). Further, reduced frontostriatal functional connectivity in response to rewards has been related to depression and specifically to anhedonia symptoms (Admon & Pizzagalli, 2015; Lichenstein, Verstynen, & Forbes, 2016). Similarly, increases in frontostriatal (nucleus accumbens with ventromedial PFC) functional connectivity in adults are positively correlated with increases in positive affect during antidepressant treatment (Heller et al., 2013).

The *structure* of the frontostriatal network has also been related to depression. A meta‐analysis (van Velzen et al., 2020) found weaker white matter microstructure (lower FA) in adults with major depression, including in the anterior limb of the internal capsule which contains part of the frontostriatal connections. However, this pattern was not related to current symptoms and not found in a smaller sample of adolescents with major depression. Frontostriatal (ventral striatum with medial PFC) structural connectivity has been positively related to trait self‐esteem in young adults (Chavez & Heatherton, 2015), and lower self‐esteem is a vulnerability factor for depression in both adults and adolescents (Sowislo & Orth, 2013). The mechanisms behind this might include lower myelination and oligodendrocyte density. Stress can affect the survival and actions of oligodendrocytes (Kokkosis, Madeira, Mullahy, & Tsirka, 2022; Orso et al., 2023), which are important for myelination and metabolic support of neurons. Also, postmortem studies have reported lower oligodendrocyte density and lower myelination in people with major depression (Hamidi, Drevets, & Price, 2004; Regenold et al., 2007; Sacchet & Gotlib, 2017; Uranova, Vostrikov, Orlovskaya, & Rachmanova, 2004).

However, there is a strong need for prospective, longitudinal studies aiming to understand the role of the frontostriatal reward network in depression in a developmental context, as pointed out by Forbes (2020). A systematic review found only six longitudinal studies on white matter structure in relation to depression in children and/or adolescents (Toenders et al., 2019). These studies had inconsistent findings, which might be the result of a lack of consideration of potential moderating or confounding variables (e.g. sex, age, pubertal development), as well as varying white matter analysis methods. Most of these studies analyzed white matter microstructure in atlas‐based tracts, meaning they did not extract frontostriatal tracts. Of these longitudinal studies, the two that did not find any prospective associations with depression symptoms also had the oldest sample, focusing on late adolescence to young adulthood. Although a lack of power or other explanations cannot be excluded, this could suggest white matter connectivity earlier in adolescence might show stronger associations with depression. Functionally, lower ventral striatum activation during reward anticipation has been linked with more depressive symptoms 2 years later, specifically for boys and girls in mid‐to‐late puberty (Morgan, Olino, McMakin, Ryan, & Forbes, 2013). However, whether the link between reward‐related structural connectivity and depression is dependent on age or pubertal development in adolescence is unknown.

Moreover, most research has focused on predicting the development of depression from brain structure or function or simple correlations between depressive symptoms and brain variables. However, depression and its functional impairments, for example, in social functioning, could impact later brain development (Pfeifer & Allen, 2021). This would partially explain the impacts on adult mental health and educational attainment described above. Several brain abnormalities in depression have been reported to be stronger in adults with recurrent depression or longer disease history (Schmaal et al., 2015; van Velzen et al., 2020), which could be an indirect indication of altered brain structure or function as a consequence of depression. Bidirectional associations, relating brain structural and functional connectivity to change in depression as well as depression to change in connectivity, have not been examined in adolescence.

Taken together, prospective, longitudinal research is needed to better understand the white matter developmental pathways that are related to the emergence of depression in adolescence. The literature points to the importance of the frontostriatal reward network but lacks examination of the coupling prospectively over time or consideration of bidirectionality. Further, unraveling the developmental differences in (potential) bidirectional associations between connectivity and depression will have important implications for the timing of prevention and intervention efforts.

### Aims and hypotheses

Therefore, we will pursue the following specific aims (preregistered at https://osf.io/fpw7y).

#### Aim 1

Establish whether associations between structural connectivity in the frontostriatal reward network and depressive symptoms in adolescent girls are unidirectional or bidirectional.

Hypothesis 1 Stronger structural connectivity in the frontostriatal reward network will be protective against the subsequent development of depressive symptoms. Further, high levels of depressive symptoms will be negatively associated with subsequent development of connectivity.

#### Aim 2

Determine to what extent the direction and strength of associations between structural connectivity and depressive symptoms are dependent on age or pubertal stage.

Hypothesis 2 The association between depression and subsequent change in connectivity will be strongest in early adolescence, leading to stronger bidirectionality of associations during early adolescence as compared with mid‐late adolescence.

## Methods

### Participants and study design

About 174 female adolescents were recruited for this longitudinal study from schools and other community sources in Oregon, USA between 2015 and 2017. Inclusion criteria at enrollment included age 10.0–13.0 years; fluent in English; normal or corrected‐to‐normal vision; no developmental disability, autism, psychotic disorder, or behavioral disorder (ODD/CD); no MRI contraindications; not (suspecting to be) pregnant; and no current use of psychotropic medication other than stimulants (amphetamines and (dex)methylphenidate). Note that we refer to participants as female/girls as they were assigned female at birth, though some identify differently (Barendse et al., 2020). For the protocol and detailed demographics see Barendse et al. (2020).

In this project, four time points were used, each roughly 18 months apart so that the total age range spanned is ages 10 through 17. The final data included 596 observations from 174 participants (T1: 174, T2: 161, T3: 148, T4: 113). See *DWI processing* for information on exclusions of scans for motion or artifacts. More information on dropout can be found in Appendix S1, Table S1.

#### Ethical information

Parents or guardians gave written informed consent and adolescents gave assent to participate. Ethics approval was granted by the University of Oregon Institutional Review Board on April 24th, 2015 (#03232015.027).

### Measures

#### Depressive symptoms

Participants reported on their depressive symptoms with the Center for Epidemiological Studies‐Depression Scale for Children (CES‐DC) (Faulstich, Carey, Ruggiero, Enyart, & Gresham, 1986; Fendrich, Weissman, & Warner, 1990) at each time point. The CES‐DC contains 20 items with responses ranging from 0 (*Not at all*) to 3 (*A lot*), with a suggested screening cut‐off of 15. The CES‐DC has excellent internal consistency and concurrent validity with the Children's Depression Inventory and DSM diagnoses, as well as good discriminant validity (Faulstich et al., 1986; Fendrich et al., 1990). The total score of the CES‐DC is used as a measure of depressive symptoms.

#### Pubertal stage

Participants completed the Pubertal Development Scale (PDS) and the Tanner Stage Line Drawings (LD) at each time point. The PDS consists of five questions regarding the adolescent's secondary sexual characteristics. We converted answers on the self‐reported PDS to Tanner Stages (Morris & Udry, 1980) using validated conversion methods (Shirtcliff, Dahl, & Pollak, 2009). The LD (Morris & Udry, 1980), female version, consists of two sets of five drawings depicting breasts and pubic hair. For both sets, adolescents choose the image that most closely reflects their current stage of development. We created a pubertal stage composite by averaging scores from the PDS and LD, which ranges from 1 (*prepubertal*) to 5 (*postpubertal*).

#### Imaging parameters

All scans were acquired on a Siemens Skyra 3.0T scanner at the Lewis Center for Neuroimaging at the University of Oregon. Participants completed a mock scan to familiarize them with the scanner at the first time point, and at every subsequent time point if time permitted. MRI scanning includes a sequence for diffusion‐weighted images with the following specifications: 64 gradient directions at *b* = 1,000 s/mm^2^ and a single nondiffusion‐weighted volume, all repeated with opposite phase encode direction; 72 slices of 2 mm isometric voxels; FOV = 208 mm; TR = 3,920 ms and TE = 75.4 ms; multiband acceleration factor = 2.

### Analyses

#### Regions of interest (ROIs)

Based on the existing evidence base described in the introduction, we focused on the following frontostriatal connections: nucleus accumbens and caudate at the striatal side to OFC and medial prefrontal cortex on the frontal cortex side. These regions of interest were defined with the Brainnetome atlas in MNI space (Fan et al., 2016). The ventral caudate (vCA) and nucleus accumbens (NAc) parcels were combined into a ventral striatal ROI. The ventromedial prefrontal cortex (vmPFC) ROI contained parcel A32sg, and the OFC ROI parcel A13 and A11l. This atlas and these parcels were chosen because of their overlap with clusters demonstrated to be part of the valuation system and clusters whose activation or connectivity has been associated with depression (Bartra, McGuire, & Kable, 2013; Chavez & Heatherton, 2015; Ng et al., 2019). Separate ROIs for each hemisphere were made and within hemisphere connectivity was quantified.

#### DWI processing

Susceptibility induced distortions were corrected using topup in FSL v6.0.1. FSL's eddy was used to correct for eddy current distortions, signal dropout and inter‐ and intra‐volume head motion (Andersson et al., 2017; Andersson, Graham, Zsoldos, & Sotiropoulos, 2016). Scans with more than 4% of volumes showing root‐mean‐square motion of >1.5 mm and scans with more than 2% outlier slices were excluded from further analyses (*n* = 11). Two scans were excluded for metal‐related artifacts. A dual fiber model was set up in BEDPOSTx (Behrens, Berg, Jbabdi, Rushworth, & Woolrich, 2007) to account for uncertainty due to crossing fibers. Probabilistic tractography between ROIs was run with PROBTRACKX (Behrens et al., 2007), with 5,000 streamlines per voxel and one of the striatal ROIs and one of the frontal ROIs as seed masks. An exclusion mask of the opposite hemisphere is used to exclude streamlines that cross hemispheres. This leads to four tracts per scan: left and right ventral striatum to vmPFC and left and right ventral striatum to OFC. We extracted the average fractional anisotropy (FA) across the tracts, weighted by the streamline density.

#### Statistical model aim 1

We initially planned to conduct Bivariate Dual Change Score Modeling (B‐DCSM, see https://osf.io/fpw7y), but these models did not converge or showed inadequate fit (see Appendix S2, Table S2, and Figure S1 for details). Therefore, we defined linear mixed models, designed to align as much as possible to the B‐DCSM, fit with restricted maximum likelihood using lmerTest in R v4.4.0. First, we conducted multiple imputation using Amelia in R v4.4.0, including all connectivity variables, depression, age and pubertal stage; we accounted for potential linear time effects in the imputation; and created 50 imputations. Conducting multiple imputation on the individual time points allowed us to use as much information as possible, as the next step was to calculate difference scores. Difference scores from each time point to the next (i.e., three difference scores for four time points) were calculated for both change in depressive symptoms and change in connectivity on all imputed data. Then we ran linear mixed models on all 50 imputations and merged the resulting coefficients using Rubin's rules (Rubin, 1987), which takes into account the uncertainty of imputations. Two models had change in depressive symptoms as outcome, one with ventral striatum to OFC connectivity as predictor and one with ventral striatum to vmPFC connectivity as predictor. The other two models had depressive symptoms as a predictor and subsequent change in ventral striatum to OFC connectivity or in ventral striatum to vmPFC connectivity as outcomes. Each difference score was treated as a repeated measure in the linear mixed model and combined with the predictor of the earlier time point, for example, ventral striatum to OFC connectivity at Time 1 predicting change in depression symptoms from Time 1 to Time 2. The data included connectivity in the two hemispheres as a repeated measure, hemisphere was included as a fixed effect and a random intercept by participant ID was defined. The formulas are provided in R formula format below and in multilevel modeling format in the Appendix S3:

$$\begin{array}{l} \text{change}\_\text{depression}˜\mathrm{VS}\_\mathrm{OFC}\_\text{connectivity}\\ \;+\;\text{hemisphere}+\left(1|\mathrm{ID}\right) \end{array}$$

$$\begin{array}{l} \text{change}\_\text{depression}˜\mathrm{VS}\_\text{vmPFC}\_\text{connectivity}\\ \;+\;\text{hemisphere}+\left(1|\mathrm{ID}\right) \end{array}$$

$$\begin{array}{l} \text{change}\_\mathrm{VS}\_\mathrm{OFC}\_\text{connectivity}˜\text{depression}\\ \;+\;\text{hemisphere}+\left(1|\mathrm{ID}\right) \end{array}$$

$$\begin{array}{l} \text{change}\_\mathrm{VS}\_\text{vmPFC}\_\text{connectivity}˜\text{depression}\\ \;+\;\text{hemisphere}+\left(1|\mathrm{ID}\right). \end{array}$$

#### Statistical model aim 2

To test Hypothesis 2 on developmental trends, we used time‐varying effect models (TVEM; Lanza, Vasilenko, & Russell, 2016), which can assess whether an association varies as a function of a developmental time variable, in our case age or pubertal stage. It accounts for clustering of repeated measures within participant ID with sandwich standard errors. We used the tvem package in R, fitting a penalized b‐spline model. B‐splines tend to be most computationally stable, and automatic penalization aims to avoid overfitting. We first fitted a model with an intercept only, reflecting change in depressive symptoms. Then we added connectivity as a predictor so that the coefficient function represents the increase in depressive symptoms as a function of connectivity at a particular age or pubertal stage. The same process was repeated with change in connectivity as the outcome and depressive symptoms as the predictor. The models were run on all 50 imputations, and the resulting coefficients merged using Rubin's rules. Change in connectivity/depression was initially planned to be captured by extracting latent variable scores using the predict function of lavaan. As the B‐DCSMs did not converge or did not show adequate fit, we used difference scores instead. The two connections (ventral striatum to mPFC and to OFC) were examined separately; hemisphere was added as a time‐varying moderator but we focus on main effects. TVEM results in a nonparametric regression coefficient function, along with the corresponding 95% confidence interval, which is plotted in the results.

## Results

**Figure 1:**
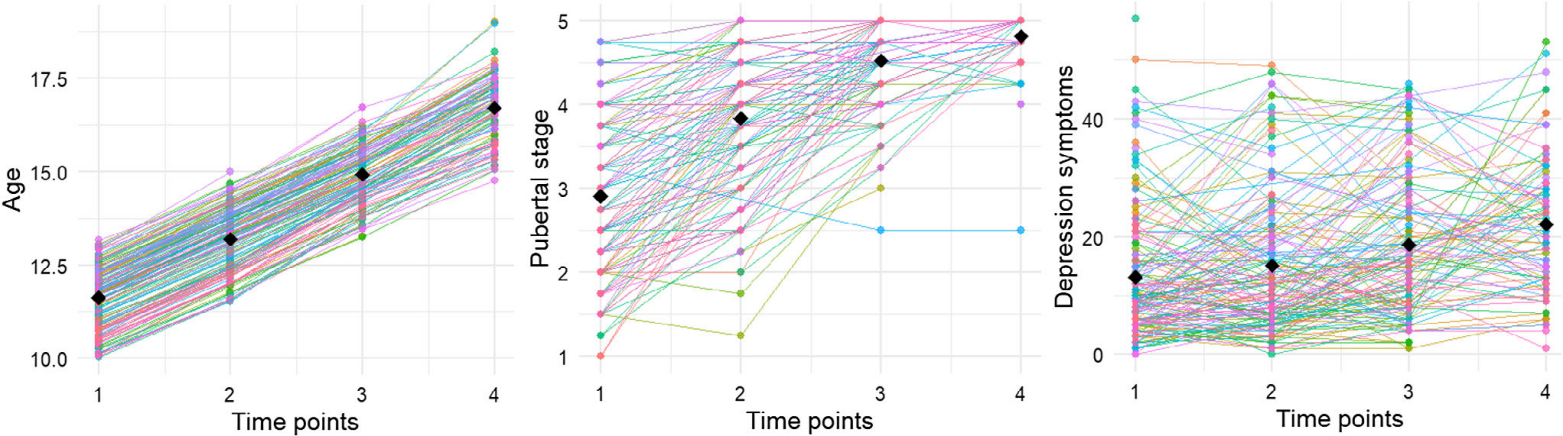
Change in age, pubertal stage and depressive symptoms across time points. The black diamond indicates the mean within each time point

**Figure 2:**
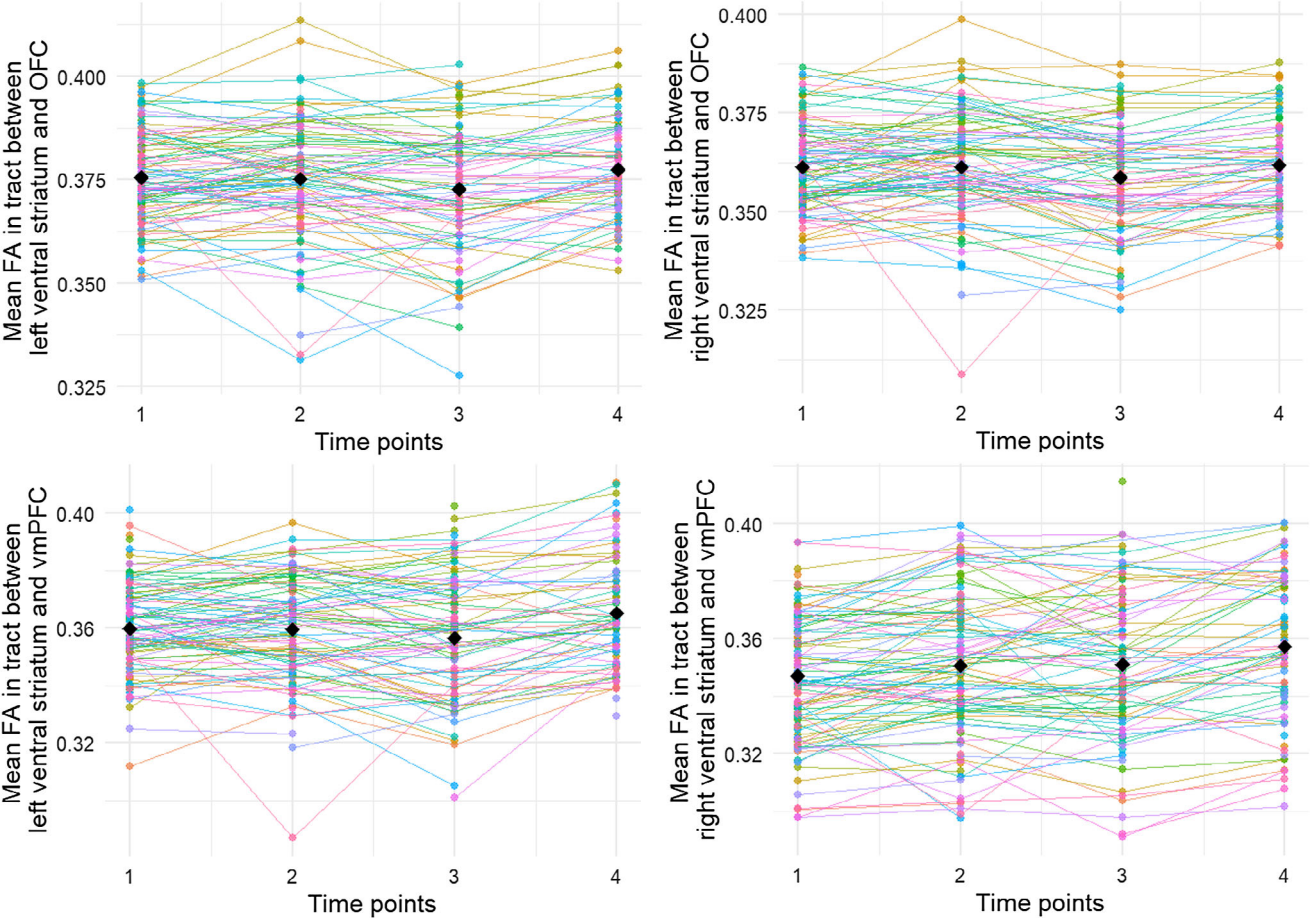
Change in frontostriatal connectivity across time points. The black diamond indicates the mean within each time point

**Figure 3:**
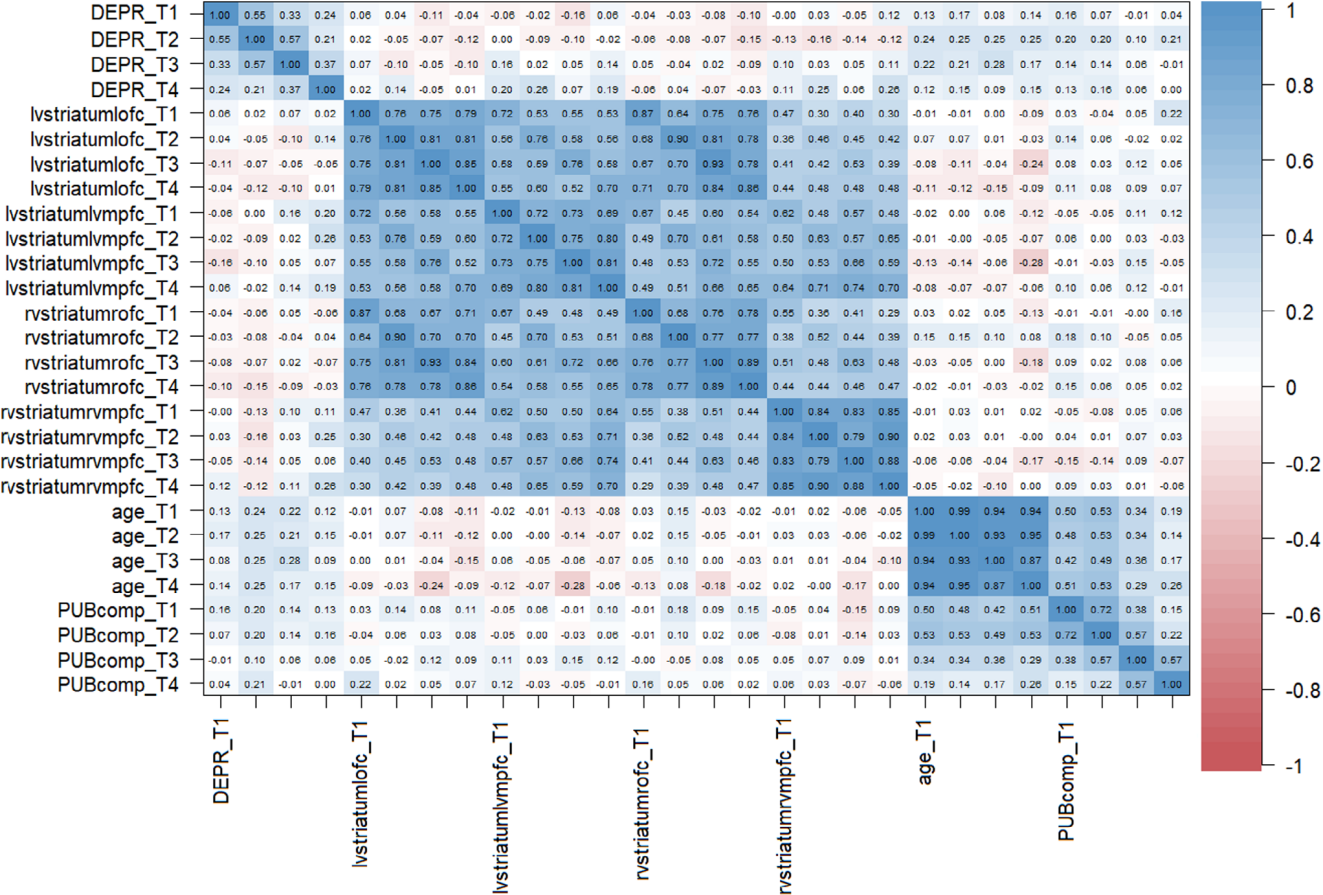
Correlations between all variables. DEPR, depression symptoms; lvstriatum, left ventral striatum; PUBcomp, pubertal stage; rvstriatum, right ventral striatum; T, timepoint

See Table 1 for descriptive information about the sample. The average level of depressive symptoms increased across time points (*F* (3,237) = 27.50, *p* < .001; Cohen's *d* from Time 1 to Time 2 = 0.18; for Time 2 to Time 3 = 0.33; and for Time 3 to Time 4 = 0.26). As shown in Table 1, the average CES‐DC score is at the screening cut‐off during Time 2 and above it for Time 3 and 4, suggesting variation extending into the clinical range. Average FA levels in the frontostriatal tracts remained stable over time. Individual trajectories are shown in Figure 1 and Figure 2. Depressive symptoms were moderately variable within individuals over time: correlations between time points were .21 to .57, see Figure 3. FA values were less variable: correlations between time points were between .68 and .90, see Figure 3. Figure 3 also shows the (cross‐sectional) correlations between depressive symptoms and connectivity measures. Adolescents who participated in all four time points did not differ in age or pubertal stage at Time 1 compared with those who did not participate in all time points, but they had fewer depressive symptoms at the initial time point (see Table S1). We reduced the impact of this by using multiple imputation, as described in the Methods.

**Table 1:** Descriptive information of the relevant variables at each time point

	Time 1 (*N* = 174)	Time 2 (*N* = 161)	Time 3 (*N* = 148)	Time 4 (*N* = 113)
Age	11.63 (0.81)	13.20 (0.84)	14.93 (0.76)	16.71 (0.86)
Pubertal stage	2.91 (0.90)	3.83 (0.80)	4.53 (0.48)	4.81 (0.34)
CES‐DC total score	13.12 (10.82)	15.07 (11.51)	18.67 (12.18)	22.13 (11.47)
FA left vstriatum – OFC	0.38 (0.01)	0.38 (0.01)	0.37 (0.01)	0.38 (0.01)
FA right vstriatum – OFC	0.36 (0.01)	0.36 (0.01)	0.36 (0.01)	0.36 (0.01)
FA left vstriatum – vmPFC	0.36 (0.02)	0.36 (0.02)	0.36 (0.02)	0.37 (0.02)
FA right vstriatum – vmPFC	0.35 (0.02)	0.35 (0.02)	0.35 (0.02)	0.36 (0.02)

Values are means with standard deviation between brackets. OFC, orbitofrontal cortex; vmPFC, ventromedial prefrontal cortex; vstriatum, ventral striatum.

Frontostriatal connectivity was not associated with subsequent change in depressive symptoms. Depressive symptoms were also not associated with subsequent change in frontostriatal connectivity. See Table 2 for details. Removing outliers (>3SD away from the mean) before imputation did not change these findings. Averaging the connectivity values across the hemispheres instead of treating hemisphere as a fixed factor also did not change the significance of the findings (note that this was a nonpreregistered sensitivity analysis).

**Table 2:** Effects of interest from the linear mixed‐effects models

Model	*b* (*SE*)	*t*	*p*	eta^2^
vstriatum – OFC to change in depressive symptoms	−11.80 (41.75)	−0.28	.78	.0005
vstriatum – vmPFC to change in depressive symptoms	17.80 (25.15)	0.71	.48	.001
Depressive symptoms to change in vstriatum – OFC connectivity	0.00003 (0.00005)	0.48	.63	.001
Depressive symptoms to change in vstriatum – vmPFC connectivity	0.00009 (0.00009)	0.95	.34	.002

To provide context to these null findings, we ran power analyses for linear mixed‐effects models using *simr* in R v.4.4.0 and found that we had 86–89% power (depending on the tested model) to detect an effect equal to an eta^2^ of .01. Therefore, we believe we had enough power to detect any effects of substantive interest.

**Figure 4:**
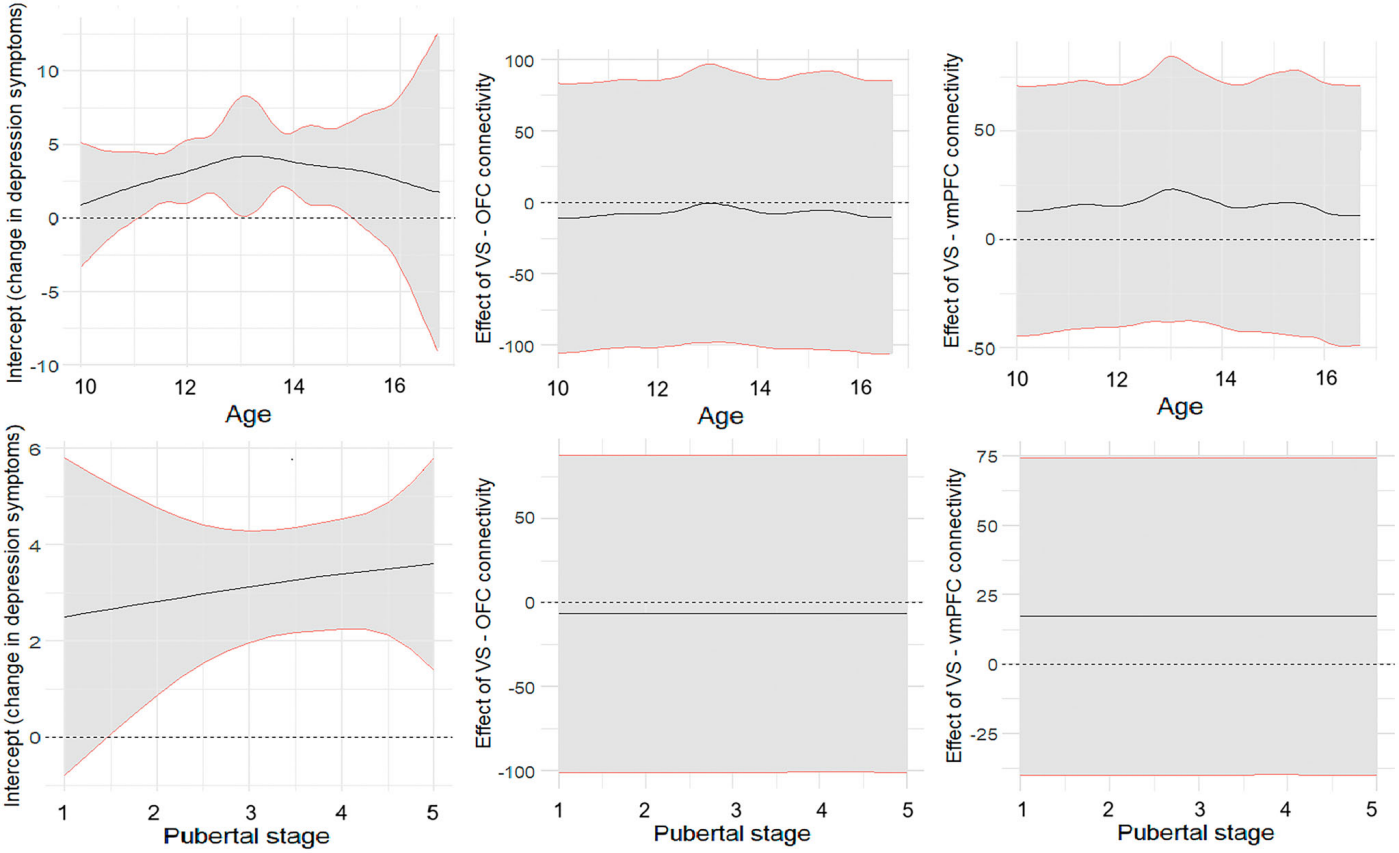
Effects of the intercept (left; age at the top, pubertal stage at the bottom), ventral striatum (VS) to orbitofrontal cortex (OFC) connectivity (middle), and ventral striatum to ventromedial prefrontal cortex (vmPFC) connectivity (right) on change in depressive symptoms

**Figure 5:**
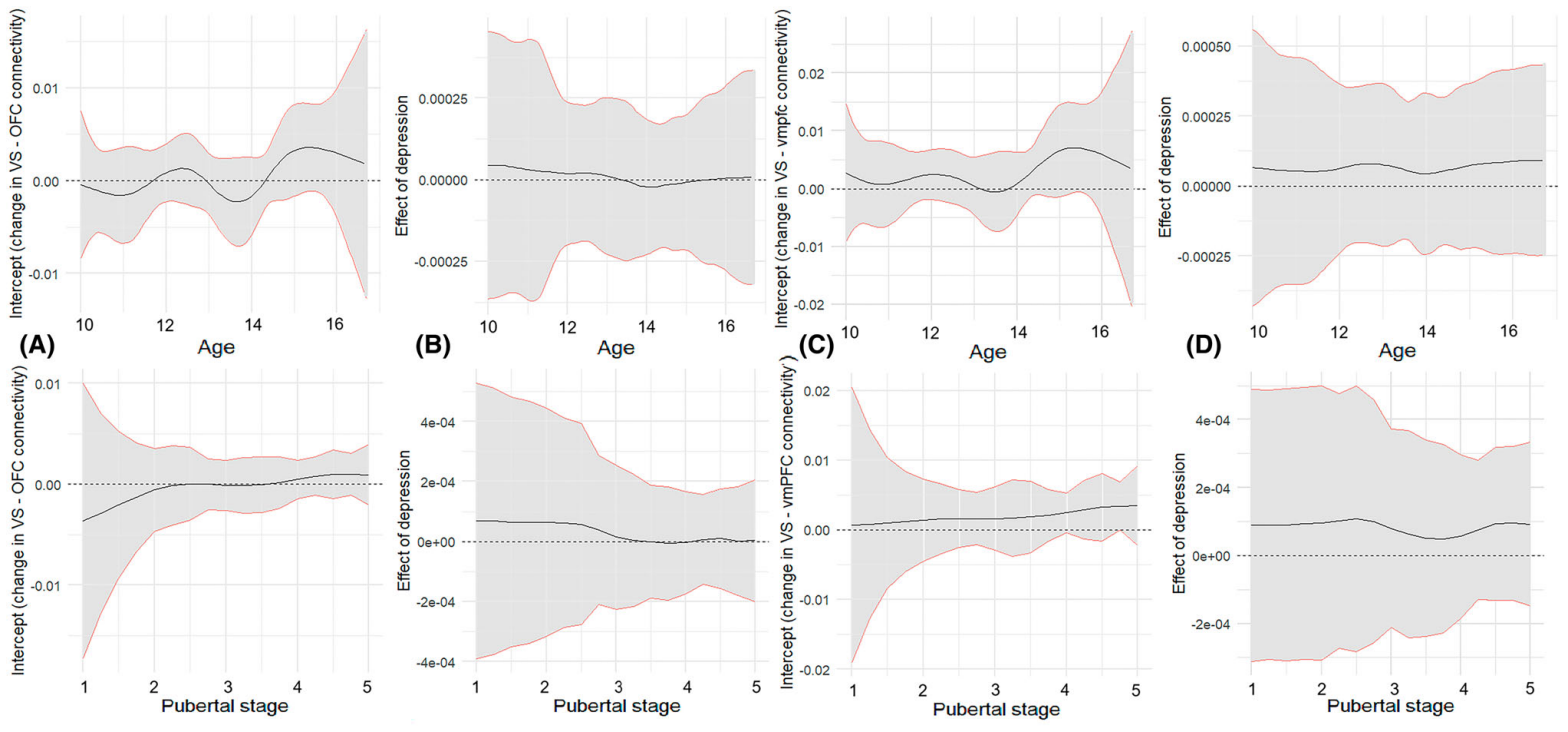
Effects of the intercept (age at the top, pubertal stage at the bottom) (A) and of depression symptoms (B) on change in ventral striatum (VS) to orbital frontal cortex (OFC) connectivity; and effects of the intercept (C) and of depression symptoms (D) on change in ventral striatum to ventromedial prefrontal cortex (vmPFC) connectivity

The analyses for aim 2 about developmental trends showed that depressive symptoms increased with age, especially in mid‐adolescence (see Figure 4). Depressive symptoms also increased with pubertal stage (not accounting for age), most strongly in the transitions to the latest pubertal stages (see Figure 4). Ventral striatum – OFC connectivity and ventral striatum – vmPFC connectivity did not have a time‐varying effect on depressive symptoms; their effect was nonsignificant at all ages and pubertal stages (see Figure 4). Depressive symptoms also did not have a time‐varying effect on change in ventral striatum – OFC connectivity or ventral striatum – vmPFC connectivity (see Figure 5).

## Discussion

In this prospective, longitudinal study, we aimed to examine bidirectional associations between structural connectivity in the frontostriatal reward network and depressive symptoms in adolescent girls. In addition, we aimed to determine to what extent the direction and strength of associations between structural connectivity and depressive symptoms are dependent on age or pubertal stage. This advances our understanding of the role of frontostriatal networks in depression in a developmental context. Unraveling the developmental differences in the associations between connectivity and depression provides important information about the ideal timing of prevention and intervention efforts. Results showed that connectivity between the ventral striatum and vmPFC or OFC was not associated with subsequent change in depressive symptoms. Depressive symptoms were also not associated with subsequent change in frontostriatal connectivity.

Previous studies on white matter connectivity in relation to depression in children and/or adolescents had been inconsistent (Toenders et al., 2019). We hypothesized that inconsistencies might in part be due to changes in the association with developmental stage, and expected the strongest effects in early adolescence. However, the association between frontostriatal connectivity and depression symptoms did not vary with age or pubertal stage. Our null findings are in line with case‐control studies. Though these studies do not specifically examine the frontostriatal network, they find lower FA in adults with depression, but not in adolescents with depression (van Velzen et al., 2020). Together, this might suggest frontostriatal structural connectivity is not a developmental mechanism prospectively related to depression in adolescent girls; and cross‐sectional associations and findings in adults with major depression might reflect other factors such as consequences of disease history or medication use over longer periods of time. Of course we have only examined ventral striatal to medial prefrontal connections, as the literature gravitates toward these pathways and they have well‐known functions in reward processing and motivation, but therefore we cannot exclude the relevance of other frontostriatal pathways for the development of depression.

It is possible that the function of the frontostriatal network is more predictive of change in depressive symptoms. The lack of average change in FA of the frontostriatal tracts as well as the high within‐person stability made it difficult to predict change over time. Function is more variable over time, and longitudinal studies have shown a consistent association between blunted striatal activation to reward and later development of depression in adolescents (Nielson et al., 2021; Toenders et al., 2019). Future research should examine prospective associations between frontostriatal functional connectivity and depression.

## Strengths, limitations, and future research

The current project is larger (4 time points, 596 observations total) than previous studies and one of the few prospective studies on frontostriatal white matter structure and depressive symptoms across adolescence. Yet, our findings have to be considered in light of several limitations. As in all longitudinal studies, there was dropout resulting in fewer observations at the oldest ages. Adolescents who participated in all four time points had fewer depressive symptoms at the initial time point. This might have reduced the overall variation in depressive symptoms or might have resulted in fewer observations of decreasing depressive symptoms (as those who start low have little room to decrease). Despite this, we had substantial variation and substantial change in depressive symptoms, as you can see in Figure 1. With null‐hypothesis testing, we cannot definitively confirm a null finding. Yet, we had enough power to detect any effects of substantive interest, as detailed in the results. Thus, we think it is unlikely that a larger sample or more time points would have led to different results. For future research we recommend a focus on other tracts or frontostriatal functional connectivity, as outlined earlier in the discussion. Further, we did not examine anhedonia specifically, since our measure of depressive symptoms (the CES‐DC) does not contain a subscale for anhedonia. Structure and function of the frontostriatal network has been linked to depressive symptoms in general and anhedonia specifically, as described in the introduction. Future research should consider separating different domains of depressive symptoms or examining a questionnaire designed to measure anhedonia. In addition, we examined moderation by pubertal stage, but other pubertal constructs, like pubertal timing, are also known to be relevant to depressive symptoms (Ullsperger & Nikolas, 2017). Pubertal timing could be examined in relation to white matter development and depression in future research. Further, we chose mixed models with change scores as our modeling strategy because it allowed us to stay as close as possible to the design of the preregistered BDCS models. However, we acknowledge that alternative strategies exist that could have fit our aims, such as cross‐lagged panel modeling. Future research could examine associations between structural connectivity and depression across adolescent development using these alternative approaches. Finally, future research should examine the same question in adolescent boys. We focused on girls due to the higher prevalence of depression in girls and the fundamental difference in pubertal development. Yet, we do not intend to and cannot generalize the findings to boys.

## Conclusion

With this study, we filled a need for prospective, longitudinal studies aiming to understand the role of frontostriatal networks in depression in a developmental context. We found that structural connectivity in the frontostriatal reward network was not associated with subsequent change in depressive symptoms. Depressive symptoms were also not associated with subsequent change in frontostriatal structural connectivity. Depressive symptoms increased with age and pubertal stage, but the association patterns did not vary with age or pubertal stage. This suggests previously reported cross‐sectional associations might not pertain to developmental effects and weakens support for the idea of frontostriatal structural connectivity as a mechanism behind the development of depression in adolescence. Future research should investigate prospective associations between frontostriatal functional connectivity and depression.

## Ethical considerations

Parents or guardians gave written informed consent and adolescents gave assent to participate. Ethics approval was granted by the University of Oregon Institutional Review Board on April 24th, 2015 (#03232015.027).

**Key points**

**What's known?**

- The frontostriatal reward network has been implicated in depression, but there is a need for prospective, longitudinal research.

**What's new?**

- We examine bidirectional associations between structural connectivity in the frontostriatal reward network and depressive symptoms in adolescent girls.

**What's relevant?**

- Frontostriatal connectivity was not associated with subsequent change in depressive symptoms and depressive symptoms were not associated with subsequent change in frontostriatal connectivity.

## Supporting information


**Appendix S1.** Dropout.
**Table S1.** Levels of depressive symptoms, pubertal stage and age at the first time point by dropout status.
**Appendix S2.** Preregistered analysis plan and results.
**Figure S1.** Illustration of the models for Aim 1, simplified for visualization purposes. lh = left hemisphere, rh = right hemisphere.
**Table S2.** Model fit (robust fit criteria) of preregistered models.
**Appendix S3.** Multilevel modeling formulas.

## Data Availability

Deidentified data of participants who gave consent for data sharing will be made available through the Research Domain Criteria (RDoC) database of the National Data Archive: https://nda.nih.gov/edit_collection.html?id=2315, reference number 2315.
