# Supplementary material for: Directionality of longitudinal associations between frontostriatal structural connectivity and depressive symptoms in adolescent girls
Source: J Child Psychol Psychiatry. 2026 Mar 5;67(8):1361–70. doi: 10.1111/jcpp.70127 (PMC13341391; doi:10.1111/jcpp.70127)
Supplement: Supplementary file 1 — Appendix S1. Dropout. Table S1. Levels of depressive symptoms, pubertal stage and age at the first time point by dropout status. Appendix S2. Preregistered analysis plan and results. Figure S1. Illustration of the models for Aim 1, simplified for visualization purposes. lh = left hemisphere, rh = right hemisphere. Table S2. Model fit (robust fit criteria) of preregistered models. Appendix S3. Multilevel modeling formulas. [file JCPP-67-1361-s001.docx]

**Directionality of longitudinal associations between frontostriatal structural connectivity and depressive symptoms in adolescent girls**

## Supporting Information

**Appendix S1. Dropout**

Participants who took part in all four time points did not differ in age or pubertal stage at time 1 compared to those who did not participate in all time points. Participants who took part in all four time points had lower depressive symptoms at time 1.

Table S1. Levels of depressive symptoms, pubertal stage and age at the first time point by dropout status

|  | Participants with 4 time points (N=107) | Participants with <4 time points (N=67) | Group differences  t (df), p |
| --- | --- | --- | --- |
| CES-DC total score (*M*) | 11.50 | 15.80 | 2.30 (99.4), .02 |
| Tanner stage (*M*) | 2.84 | 2.95 | -0.73 (126.5), .46 |
| Age(*M*) | 11.50 | 11.71 | -1.60 (134.6), .11 |

**Appendix S2. Preregistered analysis plan and results**

Our preregistered analysis plan was to implement Bivariate Dual Change Score Modeling (B-DCSM) in R to examine the bidirectional associations, thereby testing hypothesis 1. The left and right hemisphere connectivity metrics were loaded onto a latent variable representing overall connectivity. The time points were allowed to correlate within each hemisphere. Latent change variables for both connectivity and depressive symptoms were created. The regression paths between depression and change in connectivity, as well as between connectivity and change in depression, were the paths of interest (y1-3 and z1-3) in Figure S1). Ventral striatum to mPFC connectivity was modeled separately from ventral striatum to OFC connectivity. Missing data were handled with FIML, the estimator was mlr. Model fit was examined with the CFI, TLI, RMSEA, and SRMR, with fit considered adequate if it met at least three of the following four conditions: CFI ≥ 0.95, TLI ≥ 0.95, RMSEA ≤ 0.08, SRMR ≤ 0.08.

Unfortunately, these models did not converge or showed a very weak model fit (Table S2). Loosening equality constraints and other minor adjustments to the models did not improve this. Our back up model, a bivariate latent growth curve model (BLGCM), similarly showed problems with convergence and negative estimated latent variable variance. Multiple imputation of missing data (using mice in R v4.4.0 and taking the median of all imputations in order to combine the multiple imputation with the B-DCSM or BLGCM) did not resolve these issues.


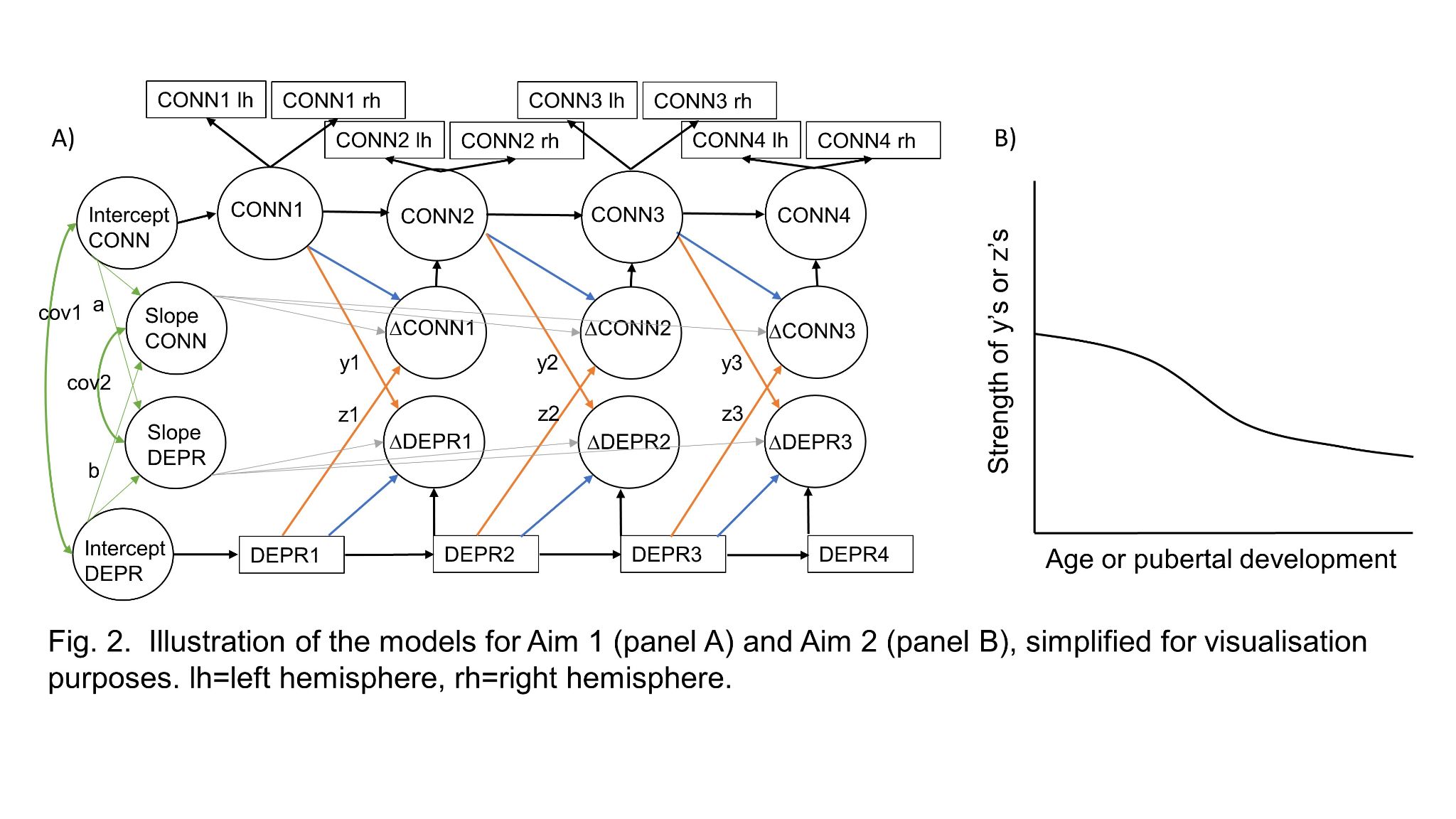


Figure S1. Illustration of the models for Aim 1, simplified for visualization purposes. lh=left hemisphere, rh=right hemisphere.

Table S2. Model fit (robust fit criteria) of preregistered models

| Model | Errors | χ^2^ | df | CFI | TLI | RMSEA | SRMR |
| --- | --- | --- | --- | --- | --- | --- | --- |
| BDCSM | | | | | | | |
| Depression & VS_OFC connectivity | Model did not converge | - | - | - | - | - | - |
| Depression & VS_vmPFC connectivity | - | 567.9 | 48 | 0.647 | 0.514 | 0.262 | 0.135 |
| BLGCM | | | | | | | |
| Depression & VS_OFC connectivity with linear slope | var-cov matrix is not positive definite, some estimated lv variances are negative (sCONN) | 1045.6 | 67 | 0.196 | 0.208 | 0.385 | 0.329 |
| Depression & VS_vmPFC connectivity with linear slope | some estimated lv variances are negative (sCONN) | 437.8 | 67 | 0.581 | 0.587 | 0.242 | 0.146 |
| Depression & VS_OFC connectivity with non-linear slope | some estimated lv variances are negative (sCONN, iCONN) | 1045.5 | 63 | 0.205 | 0.167 | 0.396 | 0.277 |
| Depression & VS_vmPFC connectivity with non-linear slope | some estimated lv variances are negative (sCONN, iDEPR) | 418.4 | 63 | 0.613 | 0.594 | 0.240 | 0.147 |

**Appendix S3. Multilevel modeling formulas**

Note: i=an observation; ID=a participant

*change_depression ~ VS_OFC_connectivity + hemisphere + (1 | ID)*

$$\Delta depression_{iID} = \beta_{0}+\beta_{1}\cdot VSOFCconnectivity_{iID}+ \beta_{2}\cdot hemisphere_{iID}+\upsilon_{0ID}+ \epsilon_{iID}$$

*change_depression ~ VS_vmPFC_connectivity + hemisphere + (1 | ID)*

$$\Delta depression_{iID} = \beta_{0}+\beta_{1}\cdot VSvmPFCconnectivity_{iID}+ \beta_{2}\cdot hemisphere_{iID}+\upsilon_{0ID}+ \epsilon_{iID}$$

*change_VS_OFC_connectivity ~ depression + hemisphere + (1 | ID)*

$$\Delta VSOFCconnectivity_{iID} = \beta_{0}+\beta_{1}\cdot depression_{iID}+ \beta_{2}\cdot hemisphere_{iID}+\upsilon_{0ID}+ \epsilon_{iID}$$

*change_VS_vmPFC_connectivity ~ depression + hemisphere + (1 | ID)*

$$\Delta VSvmPFCconnectivity_{iID} = \beta_{0}+\beta_{1}\cdot depression_{iID}+ \beta_{2}\cdot hemisphere_{iID}+\upsilon_{0ID}+ \epsilon_{iID}$$
